# 

**Published:** 1896-08-08

**Authors:** 


**Notices of Births, Deaths, and Marriages** are inserted in this column at a charge of 2s. 6d. for an announcement not exceeding four lines.

### NOTICE TO CORRESPONDENTS.

All MS., Letters, Books for Review, and other matters intended for the Editor should be addressed The Editor, The Lodge, Porchester Square, London, W.

The Editor cannot undertake to return rejected MS., even when accompanied by stamped directed envelope.

**Notice to Advertisers and Subscribers.**

All Advertisements, Orders for Copies of The Hospital and Business Communications should be addressed to **THE MANAGER, (Not to The Editor),** The Hospital, 428, Strand, London, W.C.

The Cost of Subscription, post free, is as follows:—Per week, 2½d.; per quarter, 2s. 8d.; per half-year, 5s. 3d.; per annum, 10s. 6d. Foreign:—Per Annum, 15s. 2d.; payable, in all cases, in advance.

Price 5s.

**"Burdett's Hospitals and Charities, 1896."**

Seventh year of publication. Over 1,000 pp. Scarlet cloth, gilt lettered, Price 5s. A most complete guide to British, Colonial, Indian, and American Hospitals, Dispensaries, Nursing and Convalescent Institutions, and Asylums. A few complete sets of the preceding six issues of the "Annual" may still be obtained on application to the Publishers, The Scientific Press (Limited), 428, Strand, London, W.C.

### SCRAPS AND GLEANINGS.

It is stated that the question of amalgamating the Lahore and Delhi Lunatic Asylums into one provincial asylum at Lahore is under consideration.

The Leek Memorial Cottage Hospital treated 115 patients during 1895, against 104 during 1894. The average cost per patient per week was £1 6s. 4·17d.

In 1874-5 460 cases were treated at the Monkwearmouth and Southwick Hospital. Ten years later the number of cases had risen to 2,635, and in the year ended June 30th, 1895, they had further risen to 4,150.

Five hundred and twelve in and 49 out-patients were treated at the Royal Sea Bathing Infirmary at Margate during 1895 at a total cost of £6,739. The ordinary income amounted to £6,588 and legacies to £1,242.

The Northwich and District Working Men's Hospital Saturday Committee during their eight years' existence have raised over £1,200 towards the funds of the Victoria Infirmary. Last year's collection amounted to £206.

At the West Ham Hospital, Mr. A. P. Woollright, L.S.A.Lond., has been promoted from junior to senior house surgeon, vice Mr. Hillier resigned, and Mr. J. O. Goldie, M.B., O.M.Edin., has been appointed to the vacant junior position.

Messrs. Edward Cook and Co., of the East London Soap Works, Bow, recently gave a dinner at the Holborn Restaurant to the principal members of their staff, when bonuses were announced to be distributed to those of the staff who had assisted to earn them.

Assam, with a population of 5,476,833, had a total of 97 dispensaries open on December 31st, 1895. The daily average number of in and out patients treated at these dispensaries has increased from 2,500 in 1894 to 2,741 in 1895. The total number treated was 5,987 in-patients and 538,951 out-patients.

The income of the Macclesfield Infirmary for the year 1895 compares favourably with that for the two previous years, showing a small increase on both. On the other side of the account the expenditure shows a slight decrease despite an increase of in-patients, all of which is noteworthy.

Deducting 199 readmissions, there were 671 different cases treated in the Royal Bath Hospital during 1895, of whom about 50 per cent. were cases of rheumatic and allied diseases and 40 per cent. skin diseases. Six thousand five hundred and ninety-six sulphur baths were administered.

In connection with the opening of the new pavilions at the Poplar and Stepney Sick Asylum, the question of the location of the male and female patients came under consideration, with the result that the whole of the wards on the eastern side of the administrative block is now devoted to male cases, and those on the western side to female cases.

Two hundred and twenty pounds was expended by the Bourton-on-the Water Cottage Hospital during 1895, and the receipts totalled £283; or, including a small balance of £5 in hand at the beginning of the year, £288. The balance due to the treasurer (£34) has been paid off, and a similar balance carried forward. Thirty-nine in-patients and 122 new out-patients were treated during the year.

During the last four years the number of patients treated at the West Cornwall Dispensary and Infirmary has steadily increased, as the following figures show: 1892. 105; 1893, 109; 1894, 130; and 1895, 140. This fact, added to the gradual decrease in annual subscriptions, now over £50 less than they were in 1892, makes the general tone of the last annual report of this institution a sad one.

Four thousand three hundred and eighty-two pounds was expended by the Hastings, St. Leonards, and East Sussex Hospital during 1895, and £2,748 was received (including £50 in legacies), leaving a deficit on the year of £1,634. Six hundred and twenty-one in and 2,842 out-patients were treated, rather a smaller number than usual, owing to the closing of wards for repairs.

The Association for the Oral Instruction of the Deaf and Dumb, in their report for the year 1895-6, remark concerning the work of the London School Board in the teaching of the deaf that it has not been altogether as successful as it might be. This they think arises principally from the fact that the classes for instruction are too numerous, and that therefore in one class of eight or ten children such a variety in the ages and educational status of the pupil is found that one teacher cannot successfully conduct the class. They suggest that the Board should reduce the number of its centres so that each centre should be attended by a much larger number of children, who would have to be taken thereto by rail, tram, or omnibus, or when necessary boarded out under the supervision of a boarding-out committee. Each centre would then contain a sufficient number of children to allow of their division into different classes, each class being taught by a separate teacher.

### THE STUDENT OF MEDICINE.

### APPOINTMENTS.

Dr. Banks, appointed Honorary Medical Officer to the Royal Isle of Wight Infirmary, Ryde. H. A. Caley, M.D., M.R.C.P., appointed Physician in charge of Out-patients of St. Mary's Hospital, Paddington, W. E. J. A. Chatelier, M.B., C.M.Edin., appointed Medical Superintendent of the City Hospital, Little Bromwich, Birmingham. Mary H. Cruikshank, M.D.Brux., L.R.C.P., L.R.C.S.Edin., appointed Assistant Medical Officer to the Camberwell Infirmary. Mr. N. Daly, appointed Medical Officer for the Third District of the Abingdon Union. C. H. Dyer, M.B., C.M.Aberd., appointed Medical Officer of Health to the Cleckheaton Urban District Council. R. H. Fletcher, M.R.C.S., L.R.C.P., appointed House Surgeon to the Clayton Hospital, Wakefield. A. P. Gould, M.S.Lond., F.R.C.S.Eng., appointed Surgeon in charge of the Out-patient Cancer Department of the Middlesex Hospital, W. F. W. Hall-Wright, appointed one of the Government Medical Officers to British Honduras. A. E. Hayward, M.R.C.S., L.S.A., appointed Medical Officer to the Workhouse and Second District of the Guiltcross Union. F. N. Heygate, M.R.C.S., L.S.A., appointed Medical Officer of Health to the Workhouse and the First District of the Petworth Union. A. Lawson, M.D.Brux., F.R.C.S., L.R.C.P., appointed Ophthalmic Surgeon to the Paddington Green Children's Hospital. A. W. W. Lea, M.D., B.S.Lond., F.R.C.S.Eng., appointed Assistant Physician to the Clinical Hospital for Women and Children, Manchester. S. T. Lord, M.R.C.S., L.R.C.P., appointed District Medical Officer of the Rochdale Union. J. A. Loudon, M.B., C.M., appointed Surgeon to the Coventry Provident Dispensary. R. A. Morris, M.B., B.S., M.R.C.S., L.R.C.P., appointed Medical Officer of Health to the Third District of the Morpeth Union. B. G. A. Moynihan, M.B., M.S., F.R.C.S., appointed Honorary Assistant Surgeon to the General Infirmary, Leeds. W. L. Pethybridge, M.D., B.Sc.Lond., appointed Assistant Physician to the Plymouth Public Dispensary. W. Rushton, L.D.S.R.C.S.Eng., appointed Dental Surgeon to the National Dental Hospital. D. W. Scott, L.R.C.P., L.R.C.S.Edin., appointed Medical Officer of Health to the Llanbister District of the Knighton Union. E. G. B. Starkie, M.B., C.M.Edin., appointed Medical Officer of Health to the Fylde Rural District Council. W. A. Statter, L.R.C.P.Edin., L.M., appointed Honorary Physician to the Clayton Hospital, Wakefield. G. Templeton, M.B., M.S., F.R.C.S.Eng., L.R.C.P., appointed Assistant Surgeon to the North-West London Hospital, Kentish Town Road, N.W. C. H. Whiteford, M.R.C.S., L.R.C.P., appointed Medical Officer to the Provident Branch of the Plymouth Public Dispensary. J. M. Wilson, M.D.Glasg., C.M.Camb., appointed Medical Officer of Health to the Balby with Hexthorpe Urban District Council. F. H. Wood, L.R.C.P., L.R.C.S.Edin., appointed Honorary Physician to the Clayton Hospital, Wakefield.

### PASS LISTS.

### Royal College of Physicians of Edinburgh, Royal College of Surgeons of Edinburgh and Faculty of Physicians and Surgeons of Glasgow.

The quarterly examinations for the Triple Qualification in Edinburgh took place in July, with the following results:

First Examination, Four Years' Course.—Of 13 candidates entered the following 9 passed the examination:—R. M. Hay, Yorkshire; W. M. S. Robinson, Victoria; W. E. MacManus, Longford; Lydia P. Datt, Nepal; R. J. Meade, County Cork; Amy N. DeSouza, India; W. G. Fee, New Hampshire; W. R. Jones, Bala; and A. W. Swettenham, Cheltenham.

First Examination, Five Years' Course.—Of 20 candidates entered the following 13 passed the examination:—A. F. Bowen, Shropshire; W. O. Prichard, Penmachno; J. T. Malloch, Glasgow; Winifred Muirhead, Edinburgh; G. Day, Norwich; A. Sharp, Keith; H. M. Newton, India; W. Burns, Belfast; Mary B. Carr, Eglingham; J. J. Dunne, Kilkenny; W. Welsh, Fife; C. Russell, Sutherland Durham; and J. McMurray, Warrenpoint; and 3 passed in Physics and 5 in Biology.

Second Examination, 4 Years' Course.—Of 15 candidates entered the following 8 passed the examination: H. Nuttall, Accrington; W. J. Jenner, New South Wales; M. K Quinlan, Dublin; E. H. Birchall, Oaklands; R. M. Hay, Yorks; W. R. Murison, Dundee; B. Wade, Pudsey; and W. O'Sullivan, Killarney; and one passed in Materia Medica.

Second Examination, 5 Years' Course.—Of 14 candidates entered the following 9 passed the examination: W. F. Oliver, Wark; Anne M. Watson, Edinburgh (with distinction); E. F. Cox, India; C. H. G. Lyall, Leven; R. J. Love, Castlederg; R. H. R. McKean, Chatham; G. F. Stooke, Bristol (with distinction); J. J. Dunne, Kilkenny; and J. E. Kerr, Inverness.

Third Examination, 5 Years' Course.—Of 12 candidates entered the following 11 passed the examination: Rosina J. Gillam, Brechin; Mathilda H. G. Russell, Auckland; Gertrude M. Hutton, India; Elsie R. C. Taylor, Tunbridge Wells; Flora R. C. Wood, Portobello; W. S. Soutar, Perth; E. L. Munn, Edinburgh; Margaret G. C. Brodie, Hampstead (with distinction); D. Heron, County Down; J. Hope, Edinburgh; and D. Graham, Ross-shire; and 2 passed in rnatomy and 1 in materia medica.

Final Examination.—Of 87 candidates entered the following 41 passed the examination and were admitted L.R.C.P.E., L.R.C.S.E., and L.R.P. and S.G.: H. D. Lauchlan, Bombay; J. D. McDonald Newlands, Liverpool; H. G. F. Stallard, India; L. E. Braganza, Jamnagar; F. D. Shapoorji, Surat; W. G. M. Byers, Canada; P. Heanen, Keady; E. J. Howley, Newfoundland; J. T. Waite, Halifax; R. S. Wells, Dumfries; C. E. Page, Edinburgh; A. Goltman, Montreal; M. McClelland, Ontario; W. H. G. Broyer, Melbourne; Annie L. Brennan, Bombay; R. W. Neill, Aylmer; S. Wigoder, Wiekschna; H. J. M. Wyllys, Great Yarmouth, Isabella Aitken, Berwickshire; J. S. Nedd, British Guana; S. O. Hall, India; M. Hogan, Limerick; W. Daly, Killarney; M. D. Naoroji, Bombay; A. Greer, County Armagh; A. L. de Jager, Cape Colony; C. N. F. Poole, London; M. Turkhud, Bombay; A. R. Wilkidson, Lancashire; A. J. O'Driscoll, Cork; M. F. H. Gamble, Melbourne; T. W. Pattinson, Windermere; J. J. Edgar, Daventry; S. S. Broadbent, Yorks; W. Eales, Yorks; P. B. Unwin, Wigan; J. T. Newton, Staffs; C. P. Windle, Hollin's Mount; W. H. Doran, Dublin; C. St. Hill Robinson, London; and R. Bradshaw, Sierra Leone; and 4 passed in Medicine, 1 in Surgery, and 1 in Midwifery and Medical Jurisprudence.

